# Clinical Applications of Artificial Intelligence in Structural Heart Disease

**DOI:** 10.1016/j.jscai.2026.104396

**Published:** 2026-03-25

**Authors:** Haytham Allaham, Diljon Chahal, Mukta Srivastava, Seyed Hossein Aalaei-Andabili, Anuj Gupta

**Affiliations:** Division of Cardiovascular Medicine, University of Maryland Medical Center, Baltimore, Maryland

**Keywords:** artificial intelligence, atrial fibrillation, mitraclip, structural heart disease, triclip, transcatheter aortic valve replacement

## Abstract

Transcatheter cardiac interventions have advanced substantially over the past decade, providing less invasive therapeutic options for an expanding spectrum of structural heart disease. As procedural complexity increases, artificial intelligence (AI) has emerged as a complementary tool to support clinical decision making. Early studies demonstrated that AI can automate imaging analysis, quantify cardiac anatomy, and assess hemodynamics with greater accuracy, efficiency, and reproducibility than conventional methods. This comprehensive review summarizes current clinical applications of AI in structural heart disease, focusing on its role in diagnosis, assessment of disease progression, risk stratification, procedural planning, and prediction of clinical outcomes.

## Introduction

Transcatheter heart interventions have undergone rapid evolution over the past decade, expanding from treatment of high-risk patients to broader populations with increasingly complex structural heart disease. As these procedures become more technically demanding and increasingly reliant on advanced imaging and multidisciplinary decision making, artificial intelligence (AI) has emerged as a powerful adjunct to contemporary cardiovascular care. Within this domain, machine learning (ML) and deep learning (DL) represent complementary computational approaches that enable systems to learn directly from data rather than relying on explicitly programmed rules. ML encompasses a broad class of algorithms that identify patterns and relationships within structured data, such as clinical variables, laboratory values, and quantitative imaging measurements, to support classification, prediction, and risk stratification. DL, a subset of ML, leverages multilayer neural networks capable of automatically extracting hierarchical features from high-dimensional data, including echocardiographic videos, Doppler signals, and cardiac computed tomography (CT) images, often achieving performance that rivals or exceeds expert human interpretation.

Early clinical investigations have demonstrated that AI-driven methods can automate complex imaging workflows, precisely quantify cardiac anatomy, and assess hemodynamics with greater consistency, efficiency, and reproducibility than conventional approaches. By integrating multimodal clinical and imaging data, these technologies can uncover subtle phenotypic patterns, anticipate disease progression, and inform patient-specific therapeutic strategies across the continuum of structural heart disease care. This comprehensive review synthesizes current clinical applications of AI in structural heart interventions, with a particular focus on its evolving role in diagnosis, longitudinal disease assessment, risk stratification, preprocedural planning, and postprocedural clinical outcome prediction (Central Illustration).

**Central Illustration:**
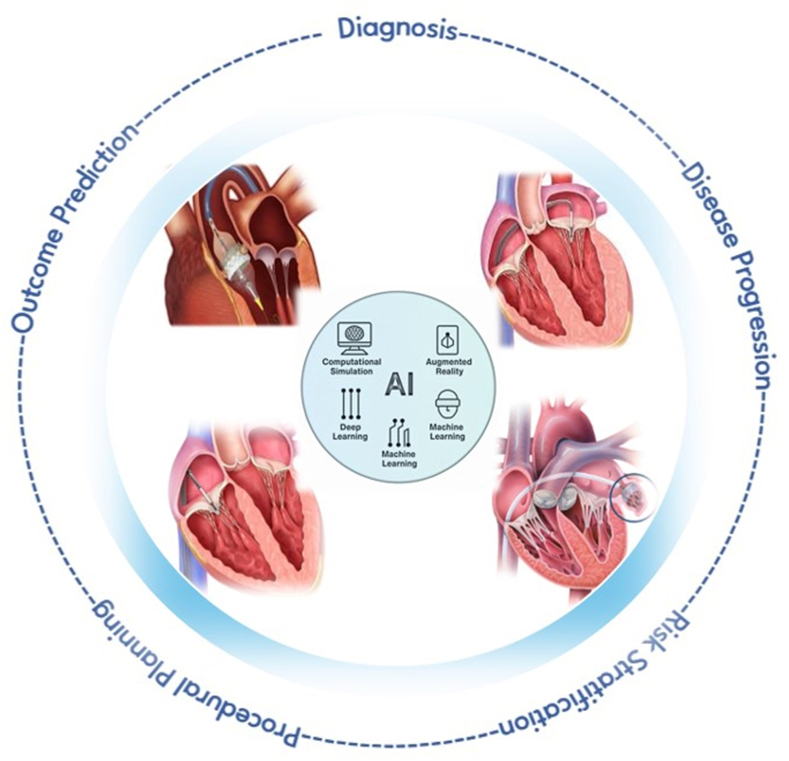
**Artificial intelligence (AI) clinical applications across the spectrum of structural heart disease.**

## The role of AI in the diagnosis of structural heart disease

Transthoracic echocardiography (TTE) remains the foundation for diagnosing and grading structural heart disease, particularly valvular pathology, yet its interpretation is inherently complex and operator dependent. AI has begun to transform this landscape by enabling automated, reproducible, and highly accurate diagnostic assessment directly from routine echocardiographic data. In aortic stenosis (AS), AI models trained on standard 2-dimensional and Doppler TTE inputs can quantify core hemodynamic parameters, including peak aortic velocity, mean transvalvular gradient, aortic valve area, and stroke volume index, in excellent agreement with expert manual measurements across the full spectrum of disease severity.^1^ By learning global imaging patterns rather than relying on isolated measurements, these systems not only replicate expert interpretation but also reduce susceptibility to technical errors that commonly affect conventional workflows. Fully automated approaches that identify severe AS independent of left ventricular outflow tract measurements have demonstrated near-complete sensitivity for high-gradient disease, highlighting the ability of AI to recognize severe phenotypes based on integrated echocardiographic features.^2^ Importantly, this capability extends to diagnostically challenging scenarios such as low-flow, low-gradient AS, where AI-based TTE analysis has shown high accuracy in distinguishing truly severe disease from moderate stenosis, potentially reducing the need for stress testing or invasive hemodynamic assessment.^3^

A similar paradigm shift is emerging in the evaluation of mitral regurgitation (MR), where diagnosis and grading traditionally require integration of multiple qualitative and quantitative parameters and are prone to interobserver variability. AI-driven echocardiographic systems can now analyze comprehensive multi-view TTE data sets, including color Doppler information, to automatically classify MR severity with performance comparable to that of expert cardiologists.^4^ Large-scale, multicenter studies have demonstrated strong agreement with expert interpretation and high discriminatory power for clinically significant regurgitation, while markedly reducing analysis time.^5^ Notably, discrepancies between AI and human readers often reveal underlying human misclassification, underscoring the potential role of AI not only as a diagnostic aid but also as a quality assurance and educational tool. By standardizing MR assessment across operators and institutions, these technologies offer a pathway toward more consistent clinical decision making and improved procedural planning.

The diagnostic challenges are even greater for tricuspid regurgitation (TR), where complex right-sided anatomy, suboptimal imaging windows, and load-dependent variability limit the reliability of conventional echocardiographic assessment. AI-based approaches address these limitations through fully automated, video-based analysis of entire echocardiographic studies, enabling robust identification and grading of TR across diverse patient populations and imaging conditions.^6^ DL frameworks trained on large, multicenter data sets have demonstrated high accuracy for detecting moderate and severe disease, with consistent performance across institutions and vendors. Other models have achieved strong concordance with expert grading and quantitative reference standards, such as vena contracta width and effective regurgitant orifice area, while maintaining low misclassification rates.^7^ Beyond traditional imaging, novel AI architectures have successfully quantified TR directly from continuous-wave Doppler spectra, bypassing the need for optimal imaging windows and further enhancing reproducibility.^8^

**Figure 1:**
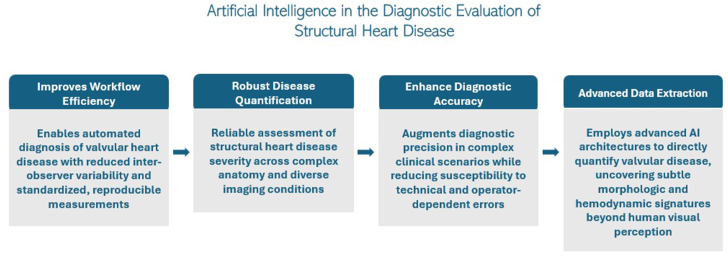
**Overview of the clinical applications and progressive integration of artificial intelligence in the diagnostic evaluation of structural heart disease.** AI, artificial intelligence.

Collectively, these advances illustrate how AI is reshaping the diagnosis of structural heart disease by extracting subtle morphologic and hemodynamic information that may be imperceptible to the human eye. By automating complex measurements, reducing variability, and maintaining high diagnostic fidelity across disease states and imaging environments, AI-enabled echocardiography holds promise for more efficient, standardized, and image-centric diagnostic strategies that may ultimately reduce reliance on invasive or adjunctive testing while improving patient care (Figure 1). Table 1^1^, ^2^, ^3^, ^4^, ^5^, ^6^, ^7^, ^8^ summarizes key clinical studies evaluating the application of AI across imaging modalities for the diagnosis of structural heart disease.

**Table 1:** The role of artificial intelligence in diagnosis of structural heart disease

Study author	Year	Clinical application	Model performance
Aortic valve stenosis			
Playford et al^2^	2020	Automated detection and classification of severe AS independent of LVOT measurement.	AUC 0.98; sensitivity 93%; specificity 91%
Krishna et al^1^	2023	Fully automated quantification of AS severity from 2D and Doppler echocardiography.	Correlation *r* = 0.97 (Vmax), 0.94 (MPG), 0.88 (AVA), 0.79 (SVi)
Wrzosek et al^3^	2026	Differentiation of true severe low-gradient AS vs moderate AS using AI-derived hemodynamic signatures	AUC 0.91; accuracy 89%
Mitral valve regurgitation			
Long et al^5^	2024	Fully automated MR classification, distinguishing primary from secondary MR and enabling comprehensive AI-based quantification.	Internal accuracy 82%; κ 0.84; AUC 0.93; external AUC 0.91
Sadeghpour et al^4^	2025	Automated MR grading improves echo lab efficiency, while 1-y mortality prediction enables timely specialist referral.	AUC 0.95 (severe MR); accuracy ∼90%
Tricuspid valve regurgitation			
Xie et al^8^	2024	Direct CW Doppler-based TR quantification bypassing imaging window constraints.	AUC 0.88 (mild), 0.84 (moderate), 0.89 (severe TR)
Vrudhula et al^6^	2025	Fully automated TR grading, enabling more precise and efficient TTE evaluation.	AUC 0.96 (severe TR); external validation AUC 0.98
Zhao et al^7^	2025	Fully automated TR grading, improving precision, reproducibility, and efficiency in TTE evaluation.	AUC 0.94 (≥ moderate TR); AUC 0.96 (severe TR)

AI, artificial intelligence; AS, aortic stenosis; AUC, area under the receiver operating characteristic curve; AVA, aortic valve area; CW, continuous wave; LVOT, left ventricular outflow tract; MPG, myocardial perfusion grade; MR, mitral regurgitation; SVi, stroke volume index; TR, tricuspid regurgitation; TTE, transthoracic echocardiogram; Vmax, peak velocity.

## The role of AI in risk stratification and prediction of disease progression

Beyond its role in diagnosis, AI is increasingly redefining how disease progression and clinical risk are understood and managed across structural heart disease. In AS, AI-driven phenotyping has demonstrated particular value in uncovering clinically meaningful heterogeneity that is not captured by conventional severity thresholds. ML decision-support models can identify subsets of patients with moderate AS who exhibit echocardiographic and clinical profiles resembling severe disease and who experience long-term mortality rates comparable to those with overt severe stenosis.^9^ These findings highlight how AI-derived phenotypes can expose “high-risk” biology within ostensibly lower-risk categories, supporting closer surveillance and potentially earlier intervention. Similarly, AI frameworks that integrate clinical and imaging variables have been shown to reclassify AS severity in patients with discordant conventional findings, offering superior prognostic discrimination and more accurate prediction of long-term outcomes such as aortic valve replacement.^10^ By moving beyond rigid guideline-based cutoffs, AI enables a more nuanced, outcome-oriented assessment of risk.

AI has also begun to address a major unmet need in AS care: prediction of disease progression over time. Echocardiography-based ML models can accurately forecast progression from mild or moderate to severe stenosis, achieving high discriminatory performance and enabling prediction at multiple time horizons.^11^^,^^12^ These tools support individualized surveillance strategies by identifying patients likely to experience rapid progression while safely reducing unnecessary follow-up imaging in lower-risk individuals. Collectively, these advances position AI as a key enabler of longitudinal disease management, facilitating earlier detection of clinically significant progression, optimizing resource utilization, and aligning monitoring intensity with patient-specific risk.

In MR, timely risk stratification is critical to prevent irreversible ventricular remodeling and preserve long-term outcomes, yet conventional imaging provides limited insight into future disease trajectories. AI-driven analysis of serial echocardiographic studies has demonstrated the ability to identify patients at heightened risk of MR progression well before advanced structural deterioration occurs.^13^ DL models applied longitudinally can stratify patients by progression risk, with those in the highest risk categories showing markedly increased likelihood of advancing to severe regurgitation. This automated and reproducible approach offers a scalable strategy for continuous surveillance and may support earlier referral for transcatheter or surgical intervention, when myocardial damage is still reversible.

Beyond predicting progression, AI has enabled discovery of prognostically distinct phenotypes in mitral valve disease.^14^ Unsupervised ML analyses of mitral valve prolapse have revealed discrete clusters that reflect progressive stages of remodeling, ventricular dysfunction, and pulmonary hypertension. These phenotypes are strongly associated with myocardial fibrosis and adverse cardiovascular outcomes, including heart failure and arrhythmias. By uncovering biologically and clinically meaningful subgroups, AI-driven phenotyping refines risk stratification beyond traditional measures of regurgitation severity and offers a framework for more personalized timing of intervention and surveillance.

In TR, where clinical risk assessment has historically been limited, ML-based survival models integrating clinical and echocardiographic variables have demonstrated superior mortality prediction compared with traditional regression approaches.^15^ These models provide more accurate prognostic stratification in patients with significant TR, a population increasingly recognized to carry substantial morbidity and mortality, and may help identify patients who could benefit from closer follow-up or emerging transcatheter therapies.

AI is also transforming risk stratification in atrial fibrillation, particularly in patients being considered for left atrial appendage occlusion (LAAO). Traditional scoring systems offer population-level estimates of thromboembolic and bleeding risk but often fail to capture individual variability. ML models that integrate clinical and echocardiographic features can more accurately predict left atrial thrombus formation and major bleeding risk, consistently outperforming established risk scores across multiple time horizons.^16^^,^^17^ These advances enable more individualized assessment of competing risks and may better inform selection of pharmacologic versus device-based stroke prevention strategies.

Extending beyond risk prediction, causal ML frameworks have been applied to estimate individualized treatment effects of LAAO compared with direct oral anticoagulation therapy.^18^ By identifying patients most likely to derive net clinical benefit from device therapy, these models move beyond average treatment effects to support truly personalized decision making.

Together, these developments illustrate how AI is reshaping disease surveillance, prognostic assessment, and therapeutic selection across structural heart disease and atrial fibrillation, shifting care toward a more dynamic, patient-specific, and outcome-driven paradigm. Table 2^9^^,^^11^, ^12^, ^13^, ^14^, ^15^, ^16^, ^17^, ^18^ summarizes key studies evaluating the role of AI in predicting disease progression and risk stratification in patients with structural heart disease.

**Table 2:** The role of artificial intelligence in risk stratification and prediction of disease progression in structural heart disease

Study author	Year	Clinical application	Model performance
Aortic valve stenosis			
Sánchez-Puente et al^12^	2023	Predicts AS progression and reduces unnecessary follow-up examinations, with simulated annual savings of −49% in Europe and −13% in United States.	Internal AUC 0.90-0.92 (1-3 y); external AUC 0.85 (all time points)
Strom et al^9^	2024	Detects severe AS phenotype and flags moderate AS with severe-like phenotype and high mortality risk; supports earlier referral.	C-statistic 0.986; sensitivity 82.2%; specificity 98.1%
Itelman et al^11^	2025	Predicts progression from mild/moderate to severe AS within 5 y for targeted surveillance and earlier intervention	AUC 0.91; accuracy 83%
Mitral valve regurgitation			
Huttin et al^14^	2023	Identified MVP subtypes with increased subclinical fibrosis and cardiac event rates, enabling early risk stratification beyond standard MR grading.	Cluster 4 (severe MR + LV dysfunction): HR 6.9 for CV events
Poterucha et al^13^	2025	Predicts MR progression and outcomes, enabling earlier detection of rapid LV remodeling and high-risk trajectories.	AUC 0.94 (MR progression); time-to-event C-index 0.91
Tricuspid valve regurgitation			
Deb et al^15^	2023	Prediction of 1- and 3-y mortality, enabling risk stratification and early identification of high-risk patients for early intervention consideration.	RSF model best: C-index 0.76 (1 y), 0.73 (3 y); Brier score 0.14 ± 0.02 (good calibration)
Atrial fibrillation			
Chaudhary et al^17^	2025	Predicts major bleeding risk in AF patients on DOACs, outperforming conventional scores.	RF AUC 0.76 (95% CI 0.70-0.81) vs HAS-BLED 0.57
Ngufor et al^18^	2025	Identify AF subgroups with differential benefit from LAAO vs DOACs, enabling personalized treatment prediction.	Balanced covariates (SMD <0.1); C-index 0.83 for event-free survival
Xiong et al^16^	2025	Predicts left atrial thrombus formation in AF to guide early anticoagulation decisions.	AUC 0.79 (95% CI, 0.67-0.88).

AF, atrial fibrillation; AS, aortic stenosis; AUC, area under the receiver operating characteristic curve; CV, cardiovascular; DOAC, direct oral anticoagulant; HR, hazard ratio; LAAO, left atrial appendage occlusion; LV, left ventricle; MR, mitral regurgitation; MVP, mitral valve prolapse; RF, random forest; RSF, random survival forest; SMD, standardized mean difference.

## The role of AI in preprocedural planning of transcatheter structural heart interventions

AI is increasingly extending its influence from diagnosis into the domain of preprocedural planning and intraprocedural optimization of transcatheter structural heart interventions. Nowhere is this more evident than in transcatheter aortic valve replacement (TAVR), where cardiac CT serves as the cornerstone of procedural planning. Although CT provides unparalleled anatomic detail, manual analysis is time-consuming and prone to interobserver variability. AI-enabled CT platforms have emerged as powerful tools to automate segmentation, standardize measurements, and dramatically accelerate preprocedural workflows.^19^, ^20^, ^21^, ^22^, ^23^ Fully automated DL algorithms can now comprehensively characterize the aortic root and valvular complex, accurately quantifying annular dimensions, coronary heights, calcification burden, and aortic angulation with near-expert precision. By reliably identifying anatomic risk features such as low coronary ostia, horizontal aorta, and extensive calcification, these systems support safer device selection and access planning while reducing analysis time from minutes to seconds.

In mitral transcatheter edge-to-edge repair (M-TEER), AI is similarly reshaping procedural planning by enabling patient-specific strategy optimization. ML models trained on imaging-based simulations can predict the functional impact of different clip positions, estimating expected MR reduction and leaflet stress before device deployment.^24^^,^^25^ This capability introduces a virtual planning environment in which alternative implantation strategies can be evaluated in advance, potentially improving procedural efficiency and reducing trial and error during live intervention. Beyond simulation, AI-driven phenotyping approaches that integrate echocardiographic and clinical data have demonstrated the ability to characterize mitral valve morphology in unprecedented detail. By capturing complex geometric relationships within the mitral apparatus, such as leaflet coaptation, tenting, and annular configuration, AI models can identify morphologic patterns associated with technical success and residual regurgitation.^26^ These insights move M-TEER planning toward a more objective, reproducible, and precision-guided process, reducing dependence on operator experience alone.

The role of AI is particularly compelling in transcatheter tricuspid interventions, where right-sided anatomy and motion present substantial imaging and planning challenges. Automated DL CT platforms can now generate comprehensive 4-dimensional models of the tricuspid annulus, right atrium, and right ventricle, producing reproducible measurements that are difficult to obtain reliably with manual analysis.^27^ AI-derived anatomic metrics have been shown to correlate with procedural complexity, technical success, and fluoroscopy time, offering clinicians objective markers to anticipate procedural difficulty and tailor strategy accordingly.^28^, ^29^, ^30^ By enabling accurate quantification of annular geometry, leaflet tethering, and right-sided chamber remodeling, AI supports informed device selection and feasibility assessment for tricuspid transcatheter edge-to-edge repair, tricuspid valve replacement, and tricuspid transcatheter valve annuloplasty. Importantly, these automated workflows maintain strong concordance with expert interpretation while substantially reducing analysis time, facilitating broader adoption in clinical practice.

AI has also been increasingly integrated in preprocedural planning LAAO. Automated CT-based workflows enable rapid and accurate characterization of appendage anatomy, facilitating device sizing and morphological classification with expert-level accuracy.^31^ Three-dimensional AI-driven heart modeling platforms further enhance procedural planning by allowing real-time adjustments and reducing reliance on transesophageal echocardiography.^32^ Comparative studies have demonstrated that AI-guided CT planning combined with intracardiac echocardiography can achieve procedural success comparable to traditional transesophageal echocardiography-based approaches, while improving workflow efficiency and minimizing sedation requirements.^33^ The clinical impact of AI-based planning has been further validated in randomized data, where patient-specific computational modeling using CT-derived “digital twins” improved procedural efficiency, reduced device repositioning and contrast use, and increased rates of complete appendage closure.^34^ These improvements translated into lower rates of device-related complications and peridevice leak, highlighting the tangible benefits of AI-guided planning on procedural and early clinical outcomes.

**Figure 2:**
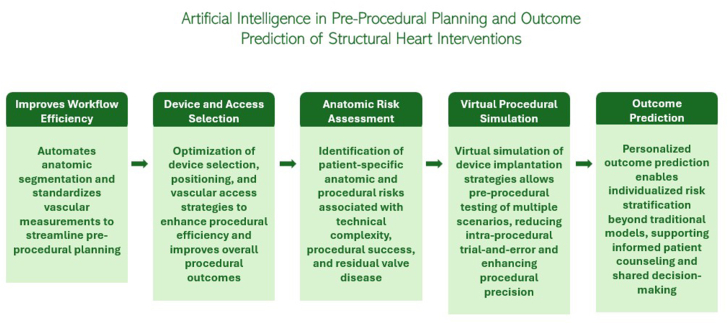
**Role of artificial intelligence in preprocedural planning for structural heart interventions, integrating automated image analysis, anatomic risk assessment, device selection, and outcome prediction to enable personalized, patient-centered decision making.**

Taken together, these advances illustrate a paradigm shift in transcatheter structural heart intervention planning. By transforming static imaging data sets into dynamic, patient-specific decision-support tools, AI enables more precise device selection, anticipatory risk assessment, and optimized procedural strategies. As these technologies continue to mature and undergo prospective validation, their integration into routine workflows has the potential to standardize complex interventions, enhance safety and efficiency, and move transcatheter therapies toward a truly precision-guided era (Figure 2). Table 3^19^, ^20^, ^21^, ^22^, ^23^, ^24^, ^25^, ^26^, ^27^, ^28^, ^29^, ^30^, ^31^, ^32^, ^33^, ^34^ summarizes key studies evaluating the utilization of AI in preprocedural planning of transcatheter structural heart interventions.

**Table 3:** The role of artificial intelligence in preprocedural planning in transcatheter structural heart interventions

Study author	Year	Clinical application	Model performance
Transcatheter aortic valve replacement			
Wang et al^19^	2023	Fully automates CT segmentation and high-risk anatomy detection, enhancing precision and reducing operator dependency in TAVR planning.	ICC up to 0.998; accuracy 0.989; sensitivity 0.979; specificity 0.986; processing time reduced >80%
Corbin et al^20^	2024	Provides expert-level CT sizing and coronary height estimation, improving accuracy and reproducibility while minimizing manual input.	Mean error <2 mm; correlation *r* ≥ 0.95; expert-level accuracy except coronary height (error ∼5%)
Toggweiler et al^21^	2024	Fully automated 2D/3D AI-based CT analysis. Streamlines TAVR planning by providing rapid, standardized anatomic measurements and valve-sizing guidance.	Correlation *r* = 0.97 (annular area/perimeter), *r* = 0.85 (STJ); correct valve sizing 87%-88%; report <10 min
Arsalan et al^22^	2024	Achieves core laboratory-level accuracy and speeds TAVR sizing and risk assessment for broader clinical adoption.	ICC >0.98 vs manual core laboratory; Analysis time reduced >70%
Mahmoudi et al^23^	2025	Facilitates optimal fluoroscopic angulation selection for commissural alignment during TAVR.	Recall 99%; precision 99%; F1 0.99; mAP50 99.5%; mean tilt error 7.9° vs interobserver 3.3°
Mitral transcatheter edge-to-edge repair			
Dabiri et al^24^	2021	Enable personalized simulation and prediction of M-TEER outcomes based on patient-specific anatomy, improving procedural planning and clip strategy selection.	Prediction *R*^2^ = 0.91 for MR reduction; correctly classified effective clip positions in >85% of cases.
Dabiri et al^25^	2023	Real-time simulation of M-TEER outcomes predicting residual MR and leaflet stress, enabling rapid intraprocedural scenario testing and optimization.	MAPE MR 0.115; stress 0.231; runtime reduced from 6 h to <1 s.
Chao et al^26^	2023	Identify anatomic and functional predictors of M-TEER success and survival to enable precision patient selection.	AUC 0.79 for phenotype-based outcome prediction
Transcatheter tricuspid valve interventions			
Bartkowiak et al^27^	2024	Fully automated quantification of anatomic predictors of T-TEER success, identifying geometric features linked to residual TR and procedural failure.	AUC 0.84 (procedural success); AUC 0.81 (≥2-grade TR reduction)
Kirchner et al^28^	2024	Fully automated guide to device positioning and trajectory planning, reducing interobserver variability and planning time.	Dice coefficient 0.93; mean error <2 mm vs manual; analysis time reduced from >45 min to <3 min
Kirchner et al^30^	2024	CT workflow quantifying annulus and RCA distance for TTVA planning, enabling safe anchoring and reduced procedural risk.	ICC >0.96 (manual agreement); AUC 0.89 (RCA–annulus safety prediction)
Yang et al^29^	2025	Pre-TTVR framework automating segmentation, measurements, and RVEF estimation for rapid, reproducible right-heart assessment.	Dice coefficient 0.95; ICC >0.98 (anatomic metrics); *R* = 0.97 (RVEF); report time <2 min vs ≈30 min manual
Left atrial appendage occlusion			
De Backer et al^34^	2023	Optimizes device selection and positioning, reducing repositioning and increasing complete closure rates.	Complete closure 61% vs 44% (*P* = .03); Fewer devices (103 vs 118, *P* < .001); Fewer repositioning events (104 vs 195, *P* < .001)
Michiels et al^31^	2022	Reduces operator variability and planning time for device sizing and trajectory prediction.	Dice coefficient 0.94 ± 0.03; mean error <1.2 mm; planning time reduced 22 min → 3 min
Kumar et al^33^	2025	AI-CT with ICE guidance enhance procedural efficiency, reducing device use and recaptures, and eliminating the need for general anesthesia.	Procedural success 97.8% vs 95.9%; Recapture 0.0 vs 0.2 (*P* = .001); devices 1.0 ± 0.2 vs 1.2 ± 0.4 (*P* = .002)
Kanschik et al^32^	2025	Real-time automated sizing and fusion imaging optimized angulation and device selection, streamlining workflow.	ICC >0.95; no difference vs TEE (*P* > .20); procedure time ↓ ∼20%

AI, artificial intelligence; AUC, area under the receiver operating characteristic curve; CT, computed tomography; ICC, intraclass correlation coefficient; ICE, intracardiac echocardiography; mAP50, mean average precision at intersection over union threshold of 0.50; MAPE, mean absolute percentage error; MR, mitral regurgitation; M-TEER, mitral transcatheter edge-to-edge repair; RCA, right coronary artery; RVEF, right ventricular ejection fraction; STJ, sinotubular junction; TAVR, transcatheter aortic valve replacement; TEE, transesophageal echocardiogram; TR, tricuspid regurgitation; T-TEER, transcatheter tricuspid edge-to-edge repair; TTVA, transcatheter tricuspid valve annuloplasty; TTVR, transcatheter tricuspid valve replacement.

## The role of AI in postprocedural clinical outcome prediction

AI is increasingly shaping postprocedural risk assessment and outcome prediction after transcatheter structural heart interventions, enabling more precise and individualized prognostication than traditional risk models. In TAVR, where clinical outcomes are generally favorable, yet heterogeneous, AI-based predictive frameworks have demonstrated clear advantages in anticipating early adverse events. ML models that integrate preprocedural clinical and echocardiographic variables consistently outperform conventional surgical and clinical risk scores in predicting in-hospital and short-term mortality after TAVR.^35^, ^36^, ^37^, ^38^ Across diverse modeling approaches, these algorithms show robust and generalizable performance, reflecting their ability to capture nonlinear relationships and complex interactions that elude traditional statistical methods. Beyond mortality, DL approaches have also been applied to predict early cerebrovascular events after TAVR, a rare but devastating complication, highlighting AI’s capacity to detect subtle imaging and physiologic patterns associated with neurologic risk.^39^

Another major determinant of post-TAVR outcomes is the need for permanent pacemaker implantation, a complication driven by conduction system injury during valve deployment. AI-based models that incorporate clinical, electrocardiographic, CT, and procedural variables have demonstrated strong discriminatory power in predicting post-TAVR pacemaker requirement, consistently outperforming conventional regression-based approaches.^40^, ^41^, ^42^ Importantly, these models maintain predictive accuracy across short- and intermediate-term horizons, enabling clinicians to estimate individualized pacemaker risk before the procedure. Such insights can inform device selection, implantation strategy, and postprocedural monitoring, supporting more personalized patient counseling and efforts to minimize conduction-related complications.

Hospital readmission after TAVR remains a common and costly challenge, often reflecting heart failure decompensation, vascular complications, or rhythm disturbances. ML models derived from large administrative and clinical data sets have shown strong performance in identifying patients at elevated risk for early readmission, effectively stratifying individuals into low- and high-risk groups with markedly different 30-day readmission rates.^43^, ^44^, ^45^ By integrating routinely available variables, these tools offer a scalable approach to post-discharge risk assessment and may enable targeted follow-up strategies, early intervention, and resource optimization aimed at reducing unplanned rehospitalizations.

In the context of M-TEER, postprocedural outcome prediction is particularly important given the high-risk profile of patients undergoing this therapy. AI-driven risk models have demonstrated substantial value in refining prognostic assessment beyond existing clinical scores. ML-based tools that integrate clinical, laboratory, and echocardiographic variables can accurately predict short- and long-term mortality after M-TEER, consistently outperforming conventional risk models.^46^, ^47^, ^48^, ^49^ These frameworks enable granular stratification across a wide spectrum of risk, identifying patients with excellent expected outcomes as well as small subgroups with extremely poor prognosis in whom procedural benefit may be limited. Such stratification supports more informed patient selection, realistic expectation setting, and shared decision making.

Complementary AI approaches have further enhanced post-M-TEER risk assessment by identifying phenotypically distinct patient subgroups with differing susceptibility to cardiovascular death and heart failure hospitalization.^50^ Unsupervised ML analyses have revealed reproducible clusters that reflect varying degrees of ventricular dysfunction, comorbidity burden, and hemodynamic compromise, each associated with progressively different outcome profiles. These data-driven phenotypes provide clinically intuitive frameworks for anticipating postprocedural trajectories and tailoring follow-up intensity and medical therapy accordingly. In parallel, AI-based models have also shown promise in predicting early hospital readmission after M-TEER, outperforming traditional regression approaches and offering opportunities for targeted post-discharge management to mitigate early adverse events.^51^

AI-driven outcome prediction is also gaining traction in transcatheter tricuspid interventions, an area historically limited by sparse prognostic tools. ML survival models using a small number of routinely available preprocedural variables can stratify patients into distinct risk categories with markedly different long-term survival, achieving performance comparable to or exceeding established surgical risk scores.^52^ These interpretable frameworks demonstrate that AI can deliver accurate prognostic insight without sacrificing clinical transparency, supporting risk-benefit assessment in a complex and evolving therapeutic landscape.

Collectively, these studies underscore the expanding role of AI in postprocedural clinical outcome prediction across transcatheter structural heart interventions. By integrating multimodal clinical and imaging data, AI-driven models enable more accurate estimation of mortality, complications, and rehospitalization risk, supporting personalized postprocedural management strategies. As these tools continue to mature and undergo prospective validation, they hold the potential to enhance patient selection, optimize follow-up care, and ultimately improve long-term outcomes through more data-driven and individualized clinical decision making (Table 4).^35^, ^36^, ^37^, ^38^, ^39^, ^40^, ^41^, ^42^, ^43^, ^44^^,^^47^^,^^50^, ^51^, ^52^

**Table 4:** The role of artificial intelligence in postprocedural clinical outcome prediction

Study author	Year	Clinical application	Model performance
Transcatheter aortic valve replacement			
Hernandez-Suarez et al^36^	2019	Prediction of in-hospital mortality.	GBM AUC 0.91; random forest AUC 0.90; logistic regression AUC 0.85
Okuno et al^39^	2021	Prediction of 30-d cerebrovascular events.	AUC 0.79
Tsushima et al^40^	2021	Prediction of post-TAVR pacemaker implantation.	AUC 0.82; accuracy 69%-75%
Sulaiman et al^44^	2022	Prediction of 30- and 90-d readmission after TAVR	External AUC 0.74
Russo et al^35^	2024	Identification of modifiable predictors of excellent tier-1 outcomes	AUC 0.77; sensitivity 67%; specificity 73%
Mitral transcatheter edge-to-edge repair			
Sulaiman et al^51^	2023	Prediction of 30-d readmission after M-TEER.	Gradient boosting AUC 0.80; logistic regression AUC 0.74
Zweck et al^47^	2021	Prediction of all-cause mortality and HF rehospitalization post-M-TEER	C-index 0.81 (XGBoost-Cox) vs EuroSMR 0.72; logistic regression 0.70
D’Ascenzo et al^50^	2025	Predict 1-y cardiovascular death or HF hospitalization after M-TEER	Identified 4 risk clusters (CV death/HF 42%-37% vs 25%-20%); validation AUC 0.78
Transcatheter tricuspid valve interventions			
Fortmeier et al^52^	2025	Prediction of 2-y survival after T-TEER; identification of high-risk patients.	Comparable to TRI-SCORE; outperformed EuroScore II (NRI 0.11; *P* = .021)

AUC, area under the receiver operating characteristic curve; CV, cardiovascular; EuroSMR, European Registry of Transcatheter Repair for Secondary Mitral Regurgitation; GBM, Gradient Boosting Machine; HF, heart failure; M-TEER, mitral transcatheter edge-to-edge repair; TAVR, transcatheter aortic valve replacement; T-TEER, transcatheter tricuspid edge-to-edge repair; XGBoost-Cox, eXtreme gradient boosting with Cox proportional hazards regression.

## Future considerations

The next phase of AI integration in structural heart disease will require sustained emphasis on transparency, reproducibility, and equitable performance. This will necessitate rigorous external validation across diverse populations, standardized reporting of model development and limitations, and prospective evaluation of real-world effects on clinical decision making and patient outcomes. Ethical oversight and governance frameworks are essential to ensure that AI deployment mitigates, rather than exacerbates, disparities in care.

Beyond technical applications, emerging AI innovations are increasingly shaping clinician–patient communication. By facilitating clearer, data-driven discussions of disease trajectories, procedural risk, and expected outcomes, AI has the potential to strengthen patient understanding and shared decision making. Realizing this promise will require deliberate physician engagement, with clinicians educated as informed users of AI tools who can critically assess their performance and limitations. Such engagement ensures that AI augments clinical expertise, preserves professional accountability, and reinforces trust at the core of structural heart care.

## Conclusion

AI now permeates the structural heart disease continuum, encompassing diagnostic imaging, patient selection, procedural planning, and outcome prediction. Validated AI applications have demonstrated improvements in reproducibility, workflow efficiency, and personalization of care, addressing long-standing sources of variability in conventional practice. By integrating multimodal clinical, imaging, and procedural data, AI enables refined phenotypic characterization, anticipates disease trajectories, and informs individualized therapeutic strategies. With continued methodological rigor and responsible implementation, AI is positioned to fundamentally reshape structural heart interventions as precision-guided, data-driven therapies. Advances in procedural efficiency, risk stratification, and treatment individualization hold promise for improving outcomes while preserving clinician judgment and patient-centered decision making. The integration of AI into structural heart workflows therefore represents a substantive shift toward a more precise, standardized, and personalized model of cardiovascular care.
